# ECAP growth function to increasing pulse amplitude or pulse duration demonstrates large inter-animal variability that is reflected in auditory cortex of the guinea pig

**DOI:** 10.1371/journal.pone.0201771

**Published:** 2018-08-02

**Authors:** Victor Adenis, Boris Gourévitch, Elisabeth Mamelle, Matthieu Recugnat, Pierre Stahl, Dan Gnansia, Yann Nguyen, Jean-Marc Edeline

**Affiliations:** 1 Paris-Saclay Institute of Neurosciences (Neuro-PSI) Université Paris-Sud, Orsay, France; 2 CNRS UMR 9197, Orsay, France; 3 Université Paris-Saclay, Orsay, France; 4 INSERM UMR-S-1159, Paris, France; 5 Université Paris-VI, Paris, France; 6 Oticon Medical—Neurelec, Vallauris, France; Universidad de Salamanca, SPAIN

## Abstract

Despite remarkable advances made to ameliorate how cochlear implants process the acoustic environment, many improvements can still be made. One of most fundamental questions concerns a strategy to simulate an increase in sound intensity. Psychoacoustic studies indicated that acting on either the current, or the duration of the stimulating pulses leads to perception of changes in how loud the sound is. The present study compared the growth function of electrically evoked Compound Action Potentials (eCAP) of the 8^th^ nerve using these two strategies to increase electrical charges (and potentially to increase the sound intensity). Both with chronically (experiment 1) or acutely (experiment 2) implanted guinea pigs, only a few differences were observed between the mean eCAP amplitude growth functions obtained with the two strategies. However, both in chronic and acute experiments, many animals showed larger increases of eCAP amplitude with current increase, whereas some animals showed larger of eCAP amplitude with duration increase, and other animals show no difference between either approaches. This indicates that the parameters allowing the largest increase in eCAP amplitude considerably differ between subjects. In addition, there was a significant correlation between the strength of neuronal firing rate in auditory cortex and the effect of these two strategies on the eCAP amplitude. This suggests that pre-selecting only one strategy for recruiting auditory nerve fibers in a given subject might not be appropriate for all human subjects.

## Introduction

Over the past decades, cochlear implants have become the most successful neuroprosthesis and are now largely used for restoring hearing. Yet, they are far from perfectly mimicking the processing that takes place in a normally functioning organ of Corti. Even after 50 years of successful use in hundreds of thousands of human subjects, studies continue to investigate the parameters required for improving speech intelligibility and music perception. For example, the optimal number of channels [1] and the mode of stimulation (monopolar vs. bipolar vs. tripolar; e.g. [2], [3], [4]) are still debated. Similarly, the optimal shape of the stimulating pulses is still the subject of intense research ([5], [6], [7], [8]). There are several ways to assess the efficacy of a particular strategy, or a particular set of stimulating parameters. In humans, psychoacoustic studies are relevant because they are non-invasive and provide the net output from the auditory system. Quantification of the electrically evoked compound action potential of the 8^th^ nerve [9] is also non-invasive but only provides responses from the cochlea. Whereas in human studies, the parameters available with electrical stimulation are limited to acceptable values for a particular patient, animal studies, which allow data sampling at several levels of the auditory system, can provide complementary results on the impact of stimulation strategies and stimulation parameters.

From the mid 80’s animal studies have investigated physiological responses to electrical stimulation made using simplified versions of human cochlear implants. Impressive effects have been reported, such as congenitally deaf cats that can efficiently use electrical cues for behavioral responses in parallel with the recovery of auditory cortex responses [10]. More detailed studies comparing the effects of different stimulation parameters have also been reported. For example, Bierer and Middlebrooks [11] recorded responses triggered by cochlear implant stimulation in the guinea pig auditory cortex and found that bipolar and tripolar stimulations induced more restricted cortical activation than monopolar stimulation. Similar findings have also been reported in the inferior colliculus [12].

A fundamental, albeit still debated question concerns how sound intensity should be coded in cochlear implants. The most employed strategy to mimic an increase in sound level is to increase the current-level (also called pulse amplitude) of the stimulation pulses [13]. It should be noted that several studies have shown that increasing pulse duration can improve psychophysical thresholds in human [14] and in animal [15]. Subsequently, it was found that changing pulse duration, or pulse rate, also induced modifications in perception of loudness [16]. Using a pulse duration strategy has technical limitations, because, for example, pulse durations that are too large intrinsically limit the available pulse rates to accurately code changes in the signal envelope. Also, it was reported that using pulse rate as a strategy to code loudness may not be as efficient as originally thought. Besides changing pitch sensation, it was reported that increase in pulse rate, which lowers thresholds, also decreases the most acceptable level of loudness and does not increase the number of discriminable intensities in human patients ([17], [18]). Although the pulse duration was demonstrated to be a key parameter for single neurons threshold [19], only a few studies have systematically evaluated whether this strategy could be used to code sound intensity by itself ([20], [21], [22]). Some cochlear implant manufacturers use increases either in pulse amplitude, or in pulse duration, to elicit changes in perception of loudness in clinical devices. Thus, it is crucial to determine if an increment in duration or in amplitude—with a constant charge per phase–has the same effects (or not) on physiological responses. Understanding how each of these parameters activates the nerve fibers can help improving loudness coding, for example by defining stimulation strategies that optimize the number of loudness steps.

Here, electrically evoked compound action potentials of the 8^th^ nerve (eCAP) were quantified either in chronically or in acutely implanted guinea pigs. The eCAP represents the synchronized responses of the first stage of the auditory pathway and is of interest for studying the input received by central auditory areas. We quantified the effects of increasing either the pulse amplitude, or the pulse duration, on the eCAP amplitude growth function and have determined if the dynamic range is larger with one strategy or another, as this has previously been evaluated in the study by Ramekers et al [23]. In animals with both chronic implants and those with acute implant (tested a few hours after implantation), the eCAP growth functions obtained by pooling together all the data for each stimulus parameter masked the existence of a large inter-animals variability. To determine whether the differences in eCAP amplitude observed between the two strategies had an impact on central physiological responses, the responses of auditory cortex neurons were analyzed in parallel with the eCAP, and the relative efficacy of the two strategies in activating cortical neurons was evaluated. We assumed that if a given strategy produced larger eCAP and also larger dynamic range of cortical responses, it could be expected that this strategy will promote better results at the perceptual level. In the present experiment, we used both relatively young (5 months) and old animals (29months) in an attempt to test the dependence on age of the observed effects.

## Methods

### Subjects

Pigmented Guinea Pigs (*Cavia Porcellus*) from 5 to 29 months of age (males n = 8, females n = 8) and weighing between 700 to 1150 g were used. The animals had access to food and water *ad libitum*. They had a heterogeneous genetic background and came from our own breeding colony, which is regularly checked by accredited veterinarians from the Essonne District. All experiments were conducted in accordance with the guidelines established by the European Communities Council Directive (2010/63/EU Council Directive Decree), which are similar to those described in the Guidelines for the Use of Animals in Neuroscience Research of the Society of Neuroscience. The protocol was approved by the ethical committee Paris-Sud and Centre (CEEA N°59, project 2014–29).

### Audiogram

The animals’ audiograms were determined 2–3 days before cochlear implantation by testing auditory brainstem responses (ABR) under isoflurane anaesthesia (2.5%). ABR were differentially recorded via two sub cutaneous electrodes (SC25, Neuro-Services), one on the vertex and the second behind the mastoid bone, next to the tympanic bulla. The ground electrode was placed in the neck. A dedicated interface and associated software (Otophylab/RT Lab, Echodia, France) allowed us to (i) present sounds monoaurally, in close field at specific frequencies with a miniaturized speaker (Knowles Electronics) equipped with a 17 mm polyethylene tube, that could be inserted into the animals’ ear canal, and (ii) record the voltage between the two recording electrodes. The signal was filtered (0.2–3.2 kHz, sampling rate 100 kHz) and waveforms were averaged (500–1000 waveforms depending on the stimulus intensity). The ABR thresholds (decibel SPL, dB) were determined as the lowest level (0 dB SPL = 20 μPa) at which a clear wave III could be observed in the ABR. The animals were tested with pure tones from 0.5kHz to 32kHz with octave steps (tone burst, 6 cycles at plateau, and 2 cycles for the rising and falling slope) presented to one ear at intensities ranging from 80 to -10 dB SPL. The guinea pigs used here were adult and some of them displayed modest hearing loss (20–30 dB in the worse cases) corresponding to their age ([24], [25]). However, we required a threshold of at least 35 dB at 16kHz for the animals to be included in the experiments.

### Cochlear implantation

The surgery for cochlear implantation was performed under general anesthesia induced by a mixture of Ketamine (KetaVet 1000, Bayer, 100 mg.kg-1, i.p.) and Xylazine (Rompun 2, Bayer, 20 mg.kg-1, i.p.), supplemented by lower doses (~ 0.3 to 0.5 ml of the mixed solution) when reflex movements were observed after pinching the hind paw. Each animal was initially placed in a stereotaxic frame for the first part of the surgery. A heating blanket allowed maintainance of the animal’s body temperature at 37°C. After injection of a local anesthetic (Xylocaine 2%, s.c.), the skin was opened and the temporal muscles were retracted. The skull was cleaned, dried and four stainless steel screws were threaded into burr holes in the calvarium to anchor a miniature socket embedded in dental acrylic. After removing the animal from the stereotaxic frame, the skin behind the right pinna was opened and the tympanic bulla was exposed. The bulla was opened under binocular control with a 2 mm cutting burr (mounted on a surgical drill) and the cochlea orientation was determined based on anatomical landmarks (round window). Cochleostomy was performed around 1–1.5 mm below the round window with a 0.4 mm diameter trephine, then enlarged with a 0.6 mm diameter trephine. The sterilized electrode-array was similar to that used by Oticon Medical/Neurelec (Platinium-Iridium electrodes, surface: 0.0046 mm^2^, diameter: 400 μm, inter-electrode distances measured center-to-center: 1000 μm, total length of the array 6mm) but limited to six electrodes. The electrode-array and the ground electrode, attached to the miniature socket, were secured to the dental acrylic. The ground electrode was inserted below the skin between the scapulae and the electrode-array was placed in front of the opened tympanic bulla. It was then anchored to the muscles next to the bulla with suture. The electrode-array was inserted into the right scala tympani. A visual confirmation of the number of electrodes inserted within the cochlea was made by direct observation through a binocular microscope. In all cases, four electrodes were inside the cochlea with the fifth on the edge of the cochleostomy as the array diameter (400 μm) prevented us inserting an electrode beyond the first turn and half in the guinea pig cochlea. In all cases, brief tests of the eCAP were performed immediately after surgery for each of the six electrodes to confirm the number of electrodes properly inserted in the cochlea.

For chronic animals (experiment 1), all incisions were sutured leaving only the miniature socket accessible for future eCAP tests. A general analgesic (Tolfedine 1 mg/kg, i.p.) was delivered at the end of the surgery and injections of antibiotics (Baytril, 2.5 mg/kg, i.p.) were given for five days after surgery to prevent infection. In the case of acute animals (experiment 2), the protocol was started at least one hour after insertion of the implant and lasted for 2–4 hours. Both in chronic and in acute animals, there was an increase in acoustic threshold that ranged from 20 to 40 dB in the high and mid frequencies after the cochlear implant insertion. At the end of the data collection, both acute and chronic animals were euthanized by a lethal injection of Dolethal (200mg/kg)

### Protocol and strategies of stimulation for eCAP recordings

The stimulation protocol was controlled via a board designed by Oticon Medical (Oticon Medical/Neurelec, Vallauris) and connected to the implant by the miniature socket secured on the animal’s head. The impedance of each electrode was tested before starting each stimulation protocol. Biphasic pulses in a monopolar configuration, starting by anodic first phase, were used with current return to the common ground electrode and a stimulation rate of 24 Hz. The two strategies differed by the parameter that was modulated to increase the level of injected charge.

1. The first strategy used pulse amplitude (PA) to increase the injected charges. The protocol included 20 blocks of 128 stimulations. Each block delivered a pulse of particular amplitude ranging from 100 μA to 1050 μA (increments of 50 μA between each block) with a fixed pulse duration of 30 μsec/phase and an interphase gap of 15 μsec. The total injected charge ranged from 3 nC per phase to 31.5 nC per phase.
2. The second strategy used the pulse duration (PD) to increase the injected charges. The protocol also included 20 blocks of 128 stimulations and in chronic animals each block delivered a pulse of particular duration ranging from 15 μsec to 53 μsec per phase (increments of 2 μsec per block) with a fixed pulse amplitude of 500 μA and an interphase gap of 15 μsec. The total injected charge ranged from 7.5 nC per phase to 26.5 nC per phase. For acute animals, the durations were adjusted and ranged from 6 μs to 63 μs in steps of 3 μs, with pulse amplitude at 500 μA. This modification was introduced to exactly match the injected charges (ranging from 3 nC to 31.5 nC per phase) delivered with pulse amplitude strategy.

The multiplexing capacity of the electronic chip controlling the implant allowed for recording electrical events in less than 5 microseconds after stimulation. We used the protocol described by Dillier et al., 2002 [26] as summarized below: Two inputs channels (one for the recording electrode and another one for the common ground electrode) of an Analog/Digital converter (RP2, TDT Systems) were subtracted (sample rate: 97.6 kHz) and the result was stored in real-time by a custom MatLab script. To remove the stimulation artifact and obtain the eCAP, the classical “Forward Masking” protocol was applied [27]. Briefly, this method involved subtracting signals collected in four different conditions: (i) no stimulation (N), (ii) “probe” (P), (iii) “masker-probe” (MP) and (iv) “masker” (M). The “masker-probe” stimulus was made of 2 stimulations separated by 400 μsec: the first one triggering a refractory period during which the nerve fibers were insensitive to the second stimulation. The masker and the probe were identical stimulations (same level of current) arriving 400 μsec apart after the beginning of the data acquisition. The eCAP signals were computed by as following: eCAP = P–(MP–M)–N.

The order of presentation of the two strategies was randomized: for half of the sessions the pulse duration was tested first and the order was reversed for the other half. Within a strategy, the masker, the probe and masker-probe were also randomly presented. In all but one animal, the results presented here were obtained with the most apical electrode as stimulating electrode (E0) and its closest neighbor (E1) as recording electrode. In one animal (C2), the penultimate electrode (E1) was used as the stimulation electrode and the most apical (E0) one as the recording electrode. The results obtained from this animal did not differ from the others.

In the chronic animals, data were collected once or twice a week (from 1 to 25 weeks post-surgery, see Table 1) under isoflurane anesthesia (2.5–2.0%) during 60-minute sessions, and it was systematically checked that stimulations did not produce muscular contractions. If that was the case, the pulse amplitude was reduced (despite the fact that it reduces evoked responses amplitude) to avoid muscle twitching. This was only observed in two animals: one was discarded from the group data; for the other, the two sessions during which the pulse amplitude was reduced were discarded. Between the sessions of data acquisition, animals were not electrically stimulated.

**Table 1:** Quantification of parameters analyzed on each chronic animal.

	Age	Freq	Threshold	Period	Electrode	Impedance		Threshold		Dynamic Range		eCAP slope	
	(months)	(kHz)	(dB SPL)	(weeks)	E0 = apical	(Ohm)		(nCoulomb)		(nC)		(mV/nC)	
								PA	PD	PA	PD	PA	PD
C1	13	2	60	25	E0	1st session	2856	9	14.5				
		16	0			Mean		9	14.5	13.5	8	43.3	27.5
		32	45			Last session	11251	7.5	15.5				
C2	10	2	80	17	E1	1st session	2059	9	13.5				
		16	5			Mean		7.5	12.5	9	10	31.3	26.2
		32	40			Last session	7307	6	11.5				
C3	25	2	65	15	E0	1st session	1263	6	9.5				
		16	20			Mean		6	8.5	12	11	98.3	116.8
		32	50			Last session	8491	6	8.5				
C4	5	2	25	7	E0	1st session	1604	6	9.5				
		16	-5			Mean		7.5	10.5	15	13.5	59.7	67.5
		32	25			Last session	4937	6	10.5				
C5	29	2	90	17	E0	1st session	2389	13.5	14.5				
		16	35			Mean		13.5	14.5	18	12	11	11.1
		32	80			Last session	7968	10.5	11.5				
C6	10	2	40	7	E0	1st session	2065	16.5	11.5				
		16	0			Mean		15	11.5	16.5	15	15.1	32.3
		32	30			Last session	5871	15	11.5				

Each column shows from left to right, the animal’s age (months at the time of implantation), the threshold of ABR (dB SPL) for the different frequencies tested (kHz), the period of study (weeks), the location of the stimulating electrode, the impedance measured for the stimulating electrode (Ohm), and for each strategy, the charges at threshold (nC), the size of the dynamic range (nC), and the slope of the eCAP (mV/nC) growth function after linear fitting and selection of the fit with the least residual. For chronic animals, the impedance measured for the stimulating electrode is given for the first and last sessions of recording. The mean values are based on the growth function presented in Fig 3.

### Semi-automatic quantification of the eCAP

A peak-tracking algorithm was used for eCAP quantification. First, it calculated the mean response for the 128 stimulations per block, leading to one curve for each pulse amplitude or duration. Then, for every mean curve, it searched for minima and maxima based on the signal envelope starting with the curve obtained with the largest value of amplitude or duration. When a peak was detected, the algorithm searched for the presence of the same peak in the following blocks of lower pulse amplitude or duration within a restricted temporal window (±0.05μsec). For each peak, the software calculated the wave amplitude (absolute value between Max and Min) and saved the latency of the first point. Each wave was labeled and its coordinates saved. Visual inspection was performed after this automatic quantification to confirm the correct labeling. If the waves were not correctly quantified (in less than 20% of the cases), the experimenters then took control over the algorithm and performed the quantification/labeling manually. After averaging responses to 128 electrical stimuli, any fluctuations below 25μV were not considered as physiological events as it was too close to the detection limit of the algorithm. The threshold was the first point that was significantly above the criterion of 25μV (based upon paired t-tests).

### Quantification of the eCAP growth functions

Using the injected charges (calculated by multiplying the pulse amplitude by its duration), eCAP growth functions were computed. The N1-P2 component of the eCAP was visible in 50% of our recordings and it was often masked by the residual of the stimulation artifact at the highest current levels. Therefore, the growth functions were based upon the P2-N2 wave to compare the two stimulation strategies. The growth functions allowed us to define the threshold, the value of the saturation plateau, and the dynamic range for each strategy. The threshold was defined as the first level of charge that was significantly different from the noise (by paired t-tests with p values <0.05). A saturation plateau was defined when the MatLab fitting tool indicated the presence of an inflexion point in the growth function and a value of the first derivative close to zero. The dynamic range was the range of charge between the threshold and the saturation plateau. The slope of the growth function was obtained with a first order linear regression model between the threshold and the saturation plateau. The best fit was selected with the least squares method (all the R^2^ >0.95).

### Responses of auditory cortex neurons

In the acute animals, neuronal activity was recorded in the primary auditory cortex (A1) at the same time as the eCAP. The methods and data acquisition were exactly the same as in our previous studies ([28], [29], [30], [31]). A 16-electrode array (ø: 33 μm, <1 MΩ), composed of two rows of 8 electrodes separated by 1000 μm (350 μm between electrodes of the same row), was inserted in A1 perpendicularly to the cortical surface to record multi-unit activity in layer III/IV (depth: 500/600 μm). A small silver wire (ø: 200 μm), used as ground, was inserted between the temporal bone and the dura matter on the contralateral side. The location of the primary auditory cortex was estimated based on the pattern of vasculature observed in previous studies ([32], [33], [34], [35]). The raw signal was amplified 10,000 times (TDT Medusa). It was then processed by an RX5 multichannel data acquisition system (TDT). The signal recorded from each electrode was filtered (610–10000 Hz) to extract multi-unit activity (MUA). The trigger level was set for each electrode to select the largest action potentials from the signal. On-line and off-line examination of the waveforms indicates that the MUA recorded here was made of action potentials generated by 2 to 6 neurons in the vicinity of the electrode. For each experiment, the position of the electrode array was set in such a way that the two rows of eight electrodes sample neurons responding from low to high frequency when progressing in the rostro-caudal direction (see examples of tonotopic gradients recorded with such arrays in Fig 1 of [28] and in Fig 6A of [30]). The analyses of the cortical responses included quantifying the evoked firing rate at all the charge levels for both strategies. By pooling the responses of all the electrodes exhibiting evoked responses 6 SD above spontaneous activity, we can obtain the growth function of the auditory cortex in a given animal and correlate this cortical growth function with the eCAP growth function. The spatial extent of cortical activation was computed based upon the cortical locations where the evoked firing rate was 6 SD above the spontaneous firing rate. This allows determining if the PA or the PD strategy produced broader or smaller activation of the map of the auditory cortex.

### Statistical analyses

After checking that the analyzed variables did not violate normal distributions, paired t-tests were used to compare the eCAP amplitude values obtained with the two strategies. Bonferroni corrections were used when multiple comparisons were performed. Two-way ANOVA (with repeated measures to take into account the different acquisition sessions) were performed to compare the growth functions obtained with the eCAP amplitude as a function of the injected charges and to determine if there was an interaction between the factors “charge” and “strategy”. The level of p< 0.05 was used as threshold of the significance value.

## Results

### Experiment 1: eCAP measures in chronically implanted guinea pigs

Six guinea pigs with unilateral implants were tested from one to twenty-five weeks after surgery. During each recording session, eCAP were recorded while testing the two loudness coding strategies, namely an increase of the stimulation pulse amplitude (PA) or an increase in the pulse duration (PD).

#### eCAP quantification using a semi–automated algorithm

**Fig 1:**
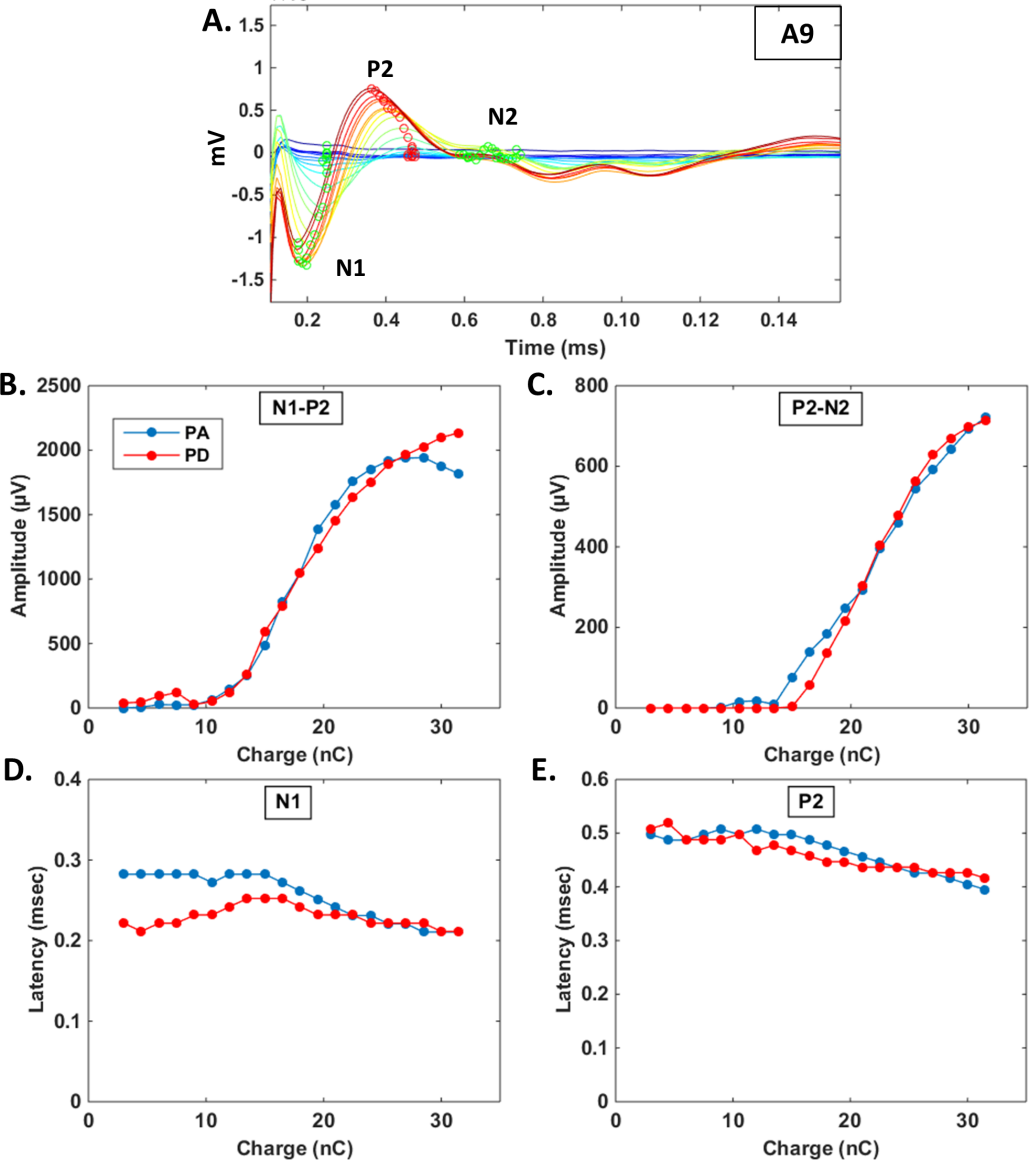
Examples of eCAP acquisition and quantification. A. Raw traces of eCAP recorded from animal A9 by increasing the pulse amplitude from blue (lowest value) to red (highest value). Each curve corresponds to the mean response averaged over 128 stimulations after removing the stimulation artifact (see Methods). The circles indicate the minimal (green) and maximal (red) values detected on each curve by the peak-tracking algorithm (see Methods). B-C. Amplitudes of the N1-P2 (B) and P2-N2 wave (C) as a function of the stimulation intensity (pulse amplitude in blue and pulse duration in red). Note that the growth functions are similar in both cases. D-E. Latency of the N1 trough (D) and of the P2 peak (E) as a function of the stimulation intensity (pulse amplitude in blue and pulse duration in red). Note that the latencies decreased as the stimulus intensity increased.

As shown from Fig 1A, after removing the stimulation artifact (see Methods) a clear physiological signal was visible, composed of a N1-P2 wave followed by a smaller N2 wave, which is in good agreement with what has been previously described in guinea pigs [36]. A custom made semi-automatic algorithm reliably allowed identification of the different eCAP waves (Fig 1A, colored circles). Fig 1B shows the N1-P2 amplitude as a function of the charges for the 2 stimulation strategies: in this particular case, there was no difference in the growth function obtained with the PA and PD strategy. Note that this holds true also for the P2-N2 wave as shown from Fig1C. Fig 1D and 1E also shows that both for the N1-P2 wave and for the P2-N2 wave, the latency regularly decreased as the charge increased, whatever the strategy was. As the N1 wave could not be systematically quantified because of its proximity with the stimulation artifact, the P2-N2 wave was used in the subsequent analyses to quantify the eCAP amplitude. The supplementary figure (S1 Fig) provides an example where the PA strategy seems to promote larger eCAP than the PD strategy both when based on the amplitude of the N1-P2 wave and based on the amplitude of the P2-N2 wave.

**Fig 2:**
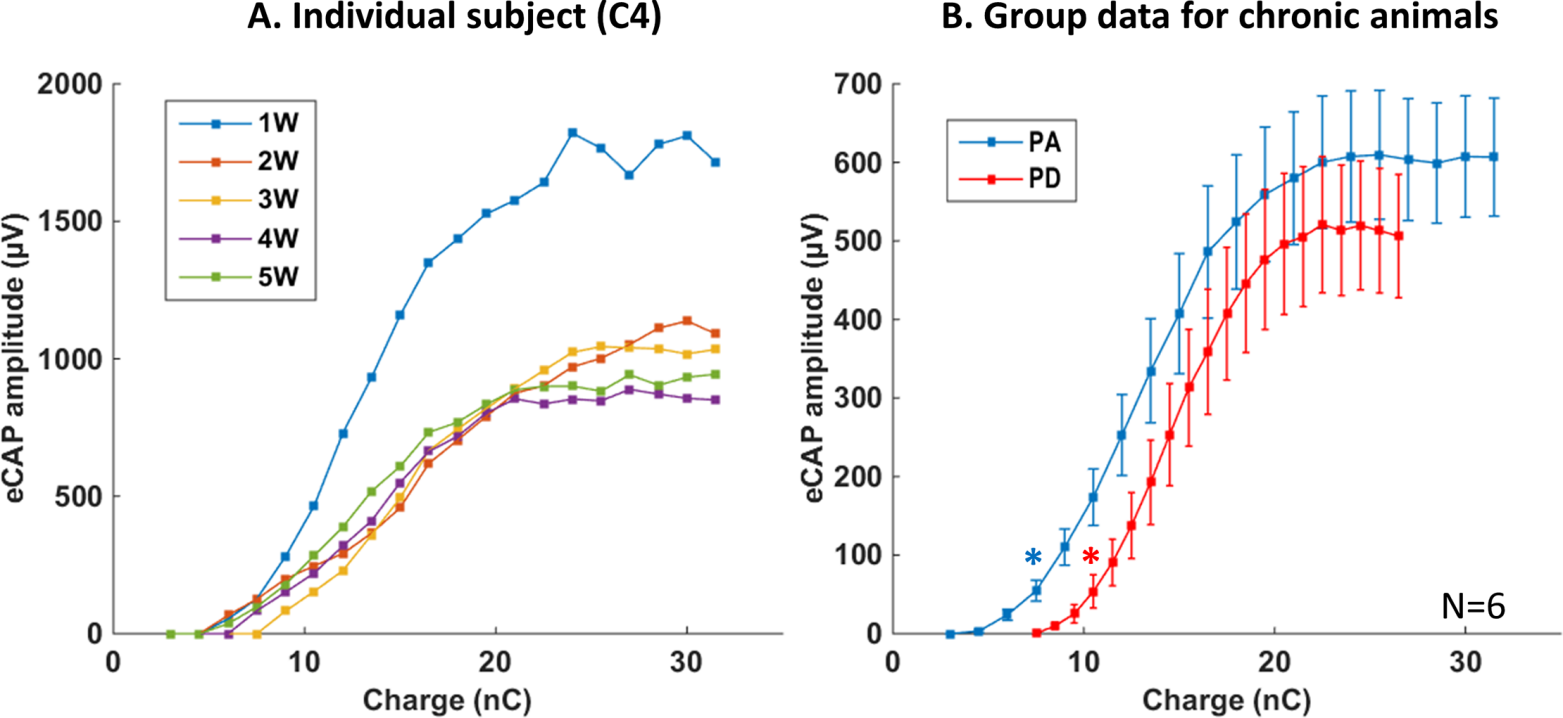
Mean growth functions for the eCAP amplitude in chronic animals. A. Amplitude of the eCAP (P2-N2 wave) as a function of the injected charges recorded in the same animal from one to five weeks after surgery with the PA strategy. Note that the eCAP amplitude largely decreased from the first to the second week post-surgery. During the subsequent weeks, it did not change significantly (p-values>0.05, paired t-test). B. Average growth function of eCAP amplitudes from all chronic animals (mean ± SEM) for the pulse amplitude (PA, blue) or pulse duration (PD, red) coding strategy. Thresholds (indicated by stars) were defined as the charge levels which triggered a response significantly larger than noise level (paired-t tests p<0.05; see Methods). The pulse duration strategy gave a higher threshold but no statistical differences were observed in the slope of the growth function and the level of the saturation plateau.

As previously described for eCAP recorded in chronically implanted guinea pigs [37], the amplitudes were relatively stable over weeks, indicating that from one recording session to the next, the automatic quantification algorithm detected similar waves. Fig 2A shows, over five weeks, the eCAP amplitude as a function of the charge level (obtained here by increasing the pulse amplitude) for one animal. Note that, after the initial difference between the first and the second week post-implantation, the growth functions of the eCAP were quite similar. In each animal, the eCAP amplitude was significantly different between the first and the second week (all p<0.02), whereas there was no statistical difference in eCAP amplitude between the second week and all subsequent weeks (all p-values > 0.05, paired t-tests).

#### Differences between group data and individual data in chronically implanted subjects

Group data for the growth function of eCAP amplitude were obtained by pooling all the sessions (except the one corresponding to the 1^st^ week) from the six implanted animals. This allowed a global direct comparison between eCAP growth function when the charge level was increased either by increasing the pulse amplitude or the pulse duration (Fig 2B). First, the eCAP threshold was higher with the PD strategy compared with the PA strategy (10.5nC vs. 7.5nC, paired t-test, p <0.05). Second, two-way ANOVA of the two growth functions (with only the iso-charge points) revealed no interaction between the factor “injected charge” and “strategy” (p = 0.53), indicating that the slopes of the growth function were not statistically different, suggesting that the auditory nerve fibers can be efficiently recruited by both strategies. Third, although the values of eCAP amplitude were systematically smaller with the PD strategy than the PA strategy, when the saturation plateau was reached there was no statistical difference between the maximal amplitude with the two strategies (lowest p value, p = 0.25, paired t-test performed for the 3 iso-charge points of the saturation plateau). This suggests that the maximum number of synchronized nerve fibers was not significantly smaller when increasing the pulse duration than increasing the pulse amplitude. The changes in eCAP latency as a function of the injected charges were similar with the PA and the PD strategy (paired t-tests, lowest p value = 0.09 for all the iso-charge points). Also, we did not observe abrupt latency shifts when increasing the charge level with one or the other strategy, suggesting there was no change in the eCAP initiation sites.

**Fig 3:**
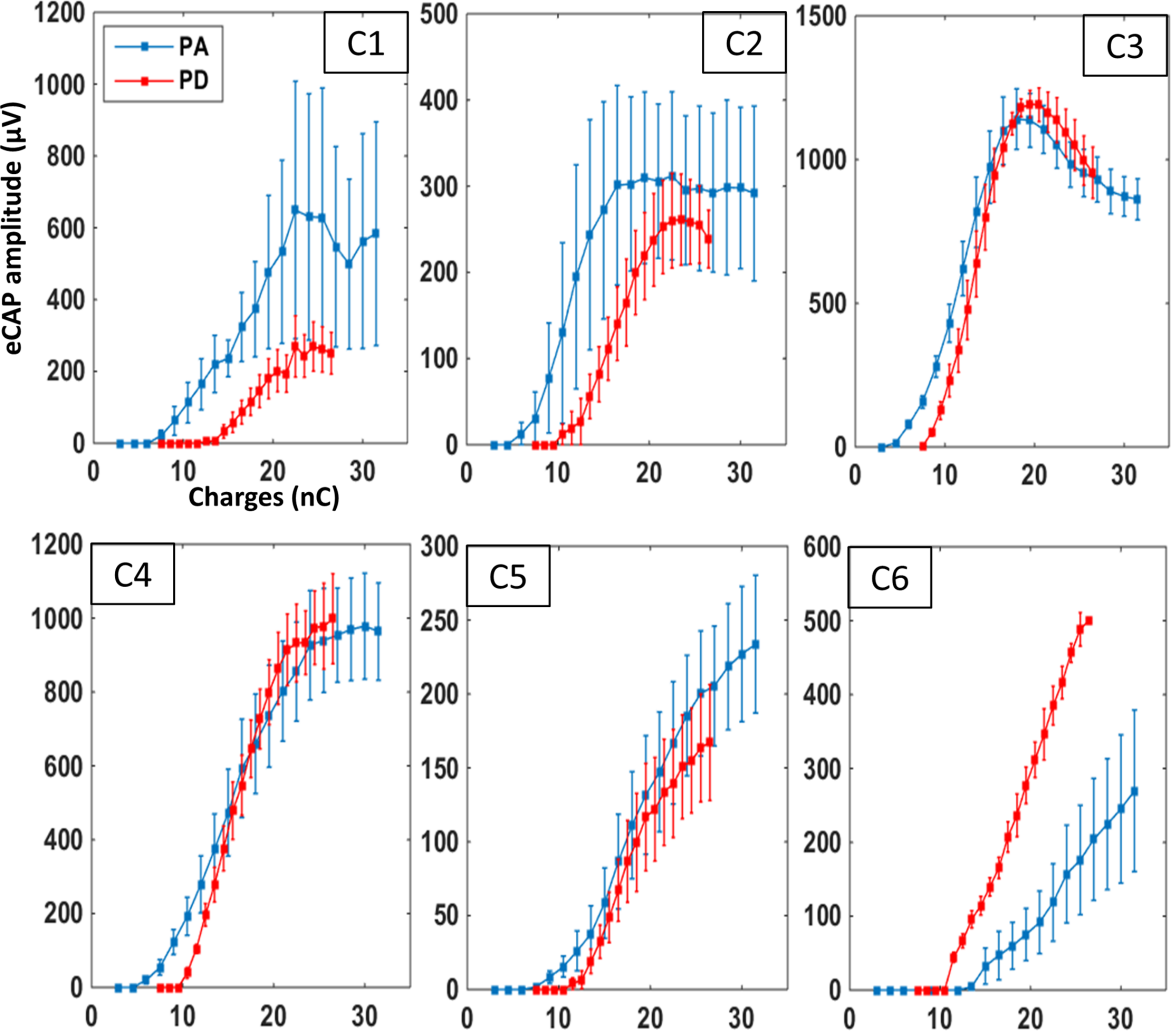
Individual mean growth functions for the eCAP amplitude in chronic animals. Each graph shows the mean (±SEM) eCAP amplitude as charge increases in the six chronic animals (C1-C6). Each curve was plotted with all the recording sessions (excluding the 1^st^ week) in a given animal. Blue curves represent eCAP amplitudes measured when charges were increased by an increase in pulse amplitude; red curves represent eCAP amplitude measured when charges were increased by an increase in pulse duration. The evolution of these two curves markedly differed from one animal to the next. Note that for some animals (C1, C2), the shape of these two curves roughly follow those shown in the group data (Fig 2B). In contrast, for other animals (e.g., C5, C6) the two curves clearly differ from the group data (Fig 2B).

Based upon these group data, the pulse duration might be considered as a less efficient strategy to code for sound intensity because it required higher thresholds than the pulse amplitude strategy. However, it is important to point out that in human subjects eCAP thresholds alone are not sufficient to predict the efficiency of a given strategy [38]. In addition, a careful examination of the individual data confirms the diversity of the eCAP growth functions. When plotting the mean growth function from each animal across all recording sessions (except that in the first week), the eCAP growth function presents striking differences from one subject to the next (Fig 3). For two animals (C1 and C2 in Fig 3), the evolution of the two growth functions was similar to that observed in the group data (Fig 2B). For two other animals (C3 and C4), the saturation plateau was similar with the two strategies and the only difference was a higher threshold with the pulse duration strategy. Lastly, in one animal (C6), the eCAP growth function was in total opposition to the group data: the threshold was lower and the eCAP amplitude was higher with the duration strategy at all charge levels. It should be kept in mind that these curves were obtained by averaging all the sessions (excluding the first week) from each animal.

We have searched for factors that could explain the difference observed from one animal to another in terms of thresholds and growth functions. Table 1 summarizes the parameters (age at time of implantation, pre-implantation acoustic threshold, post-implantation electrical threshold) observed in chronic animals (labeled C1 to C6). It shows that the lowest electrical thresholds can be seen either in relatively young (5 months for C4) or old (25 months for C3) animals. Also, the values of electrical threshold do not seem to be function of the pre-implantation acoustic threshold as the worst electrical threshold was found in an animal with a relatively good acoustic threshold (C6, but see Ramekers et al [23] for a counter-intuitive decrease in threshold when the auditory nerve degenerates). Similarly, the broadest dynamic range and the shallowest slope of the growth function were observed neither in the youngest animals nor in animals exhibiting the best auditory thresholds before implantation. We did not find any correlations between the parameters determined from eCAP growth functions and the age or pre-implantation acoustic threshold (all values of Pearson correlations <0.52; all p values >0.05).

In conclusion, from one animal to another, there was large heterogeneity in the growth functions found with the two strategies in chronic animals. Many physiological events can occur between insertion of the electrode arrays in the cochlea and the second week post-surgery, which might explain the diversity of the results. Among them, the fibrosis around the electrodes, the recovery in endolymphatic concentrations after cochleostomy (which both can change the electrode impedance) and the potential recovery from the initial damage during the insertion, can evolve differently from one animal to the next. To reduce some of the potential events explaining the diversity of our results, we ran the same protocol in another set of animals just after the insertion of the electrodes in the cochlea.

### Experiment 2: eCAP growth function during acute implantation

Ten guinea pigs with unilateral implants were tested in the 3 first hours after surgery and the eCAP growth functions were analyzed after increasing either the pulse amplitude or the pulse duration. This was performed multiple times (4–8) to guarantee the reliability of the results. The injected charges were set to have exactly the same amount of charge for every corresponding increment (for example the charge of the 10^th^ block in the pulse amplitude protocol is strictly the same than the 10^th^ block of the pulse duration protocol). This may eliminate potential bias present in the protocol performed on chronically implanted animals where only 7 points on the growth function were strictly equivalent in terms of injected charges.

#### Group data: Divergence between eCAP growth function derived from chronic and acute conditions

**Fig 4:**
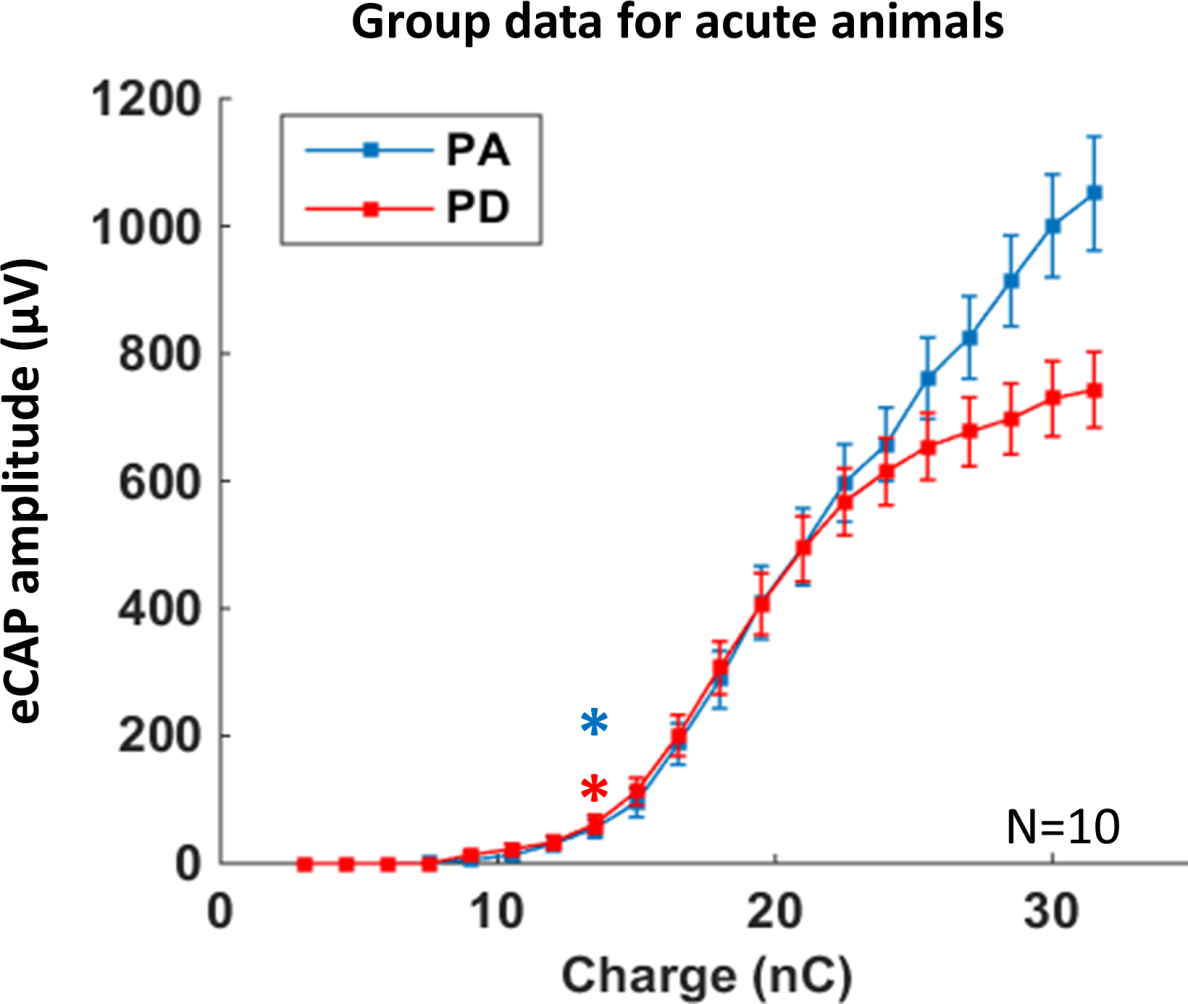
Mean growth functions for the eCAP amplitude in acute animals. Average growth function of eCAP amplitudes from the ten acute animals (mean ± SEM, 4–8 acquisition protocols for each animal) for the pulse amplitude (blue) or pulse duration (red) coding strategy. Thresholds were defined as the charge level that triggered a response significantly larger than the noise level (see Methods). Contrary to chronic animals, there was no difference in thresholds between the two strategies and a saturation plateau was only visible with the pulse duration strategy.

Fig 4 shows the average eCAP growth functions recorded in the acute animals when increasing either the pulse amplitude or the pulse duration. Two major results emerged when comparing the two growth functions. First, the eCAP thresholds (in both cases 13.5nC) and the slopes growth functions (evaluated by an ANOVA showing no interaction between “injected charge” and “strategy”, p = 0.67) were similar for PD and PA strategies. This suggests that at least for 2/3 of the charge values (the lowest), there was a similar synchronized recruitment of the auditory nerve fibers. Second, the eCAP growth function seems to reach a saturation plateau when increasing the pulse duration whereas a saturation plateau was not present when increasing the pulse amplitude. The eCAP amplitude significantly differed between the two strategies for the last four values of injected charge (paired t-tests, all p-values < 0.04).

Thus, the group data obtained from acute preparations differed from those obtained in chronically implanted animals. The difference in eCAP threshold observed in chronic animals was absent in acute animals. In contrast, the difference in terms of the saturation plateau (non-significant in chronic animals) was clear in acute animals. For 2/3 of the growth functions, the two strategies were equivalent in terms of eCAP amplitude, but the dynamic range with the PA strategy was wider than with the PD strategy (18 nC vs. 12 nC). However, as for chronic animals, examination of the growth function recorded individually for each animal revealed a large diversity.

#### Divergence between group data and individual group data for acute subjects

**Fig 5:**
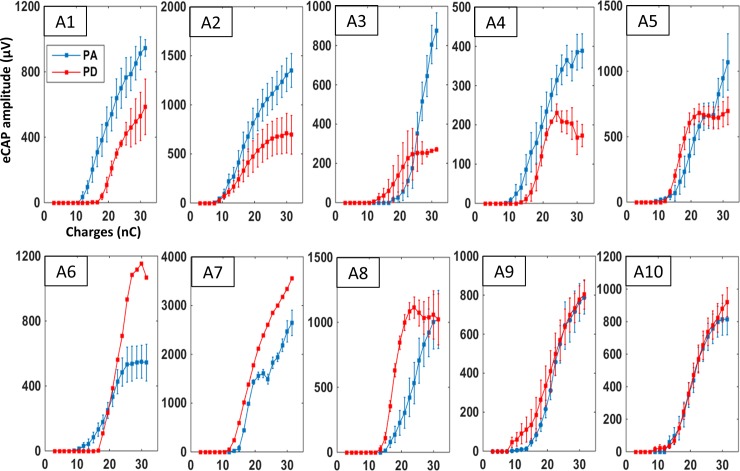
Individual mean growth functions for the eCAP amplitude in acute animals. Each graph shows the mean (±SEM) eCAP amplitude as charge increases for one of the acute animals (A1-A10). Each curve was built with 4–8 acquisition protocols in a given animal. Blue curves represent eCAP amplitudes measured when charges were increased by an increase of pulse amplitude and red curves represent eCAP amplitudes measured when charges were increased by increasing the pulse duration. The evolution of these two curves markedly differed between animals. Note that for some animals (A3, A5), the shapes of these two curves roughly follow those shown in the group data (Fig 4). In contrast, for other animals (e.g., A1, A6, A7) the two curves clearly differ from the group data (Fig 4).

The growth functions induced by the two strategies in the 10 acute animals are presented in Fig 5. As for the chronic subjects, the growth function of many animals differed from the group data and various profiles emerged. For example, for animals A1 to A5 (Fig 5, top row), it seems that the largest eCAP amplitude induced by the PD strategy was lower than with the PA strategy, even though the eCAP threshold with the PD could be either higher (A1, A4) or lower (A3, A5) than with the PA strategy. In other animals (A6, A7), the largest eCAP amplitude was obtained with the PD strategy. Note that the slope of the growth function can be either steeper (A6, A8) or smoother (A2, A3) with the PD strategy.

In the acute conditions, we also looked for factors that can explain the difference observed between animals in terms of threshold and growth function. Table 2 summarizes the parameters (age at the time of implantation, pre-implantation acoustic threshold, post-implantation electrical threshold) obtained in individual animals (labeled A1 to A10). As with the chronic animals, the lowest electrical thresholds (animal A2) were not obtained in the youngest animals or those with the best pre-implantation acoustic threshold. Also, the values of electrical threshold were not a function of the pre-implantation acoustic threshold, as the worse electrical threshold (19.5 nC) was found in an animal with a relatively good acoustic threshold (animal A3). We only found a significant negative correlation between age of the animal and the electrical threshold (r = -0.75, p<0.05) but this held true only for the PA strategy. As the nerve fibers tend to degenerate with age, this negative correlation seems to fit with the observation by Ramekers et al. [23] who found lower threshold in animals with some degeneration of the nerve fibers. We found no correlation between the eCAP max amplitude and the age of the animals for either strategies (PA r = 0.15; PD r = 0.33).

**Table 2:** Quantification of the parameters analyzed on each acute animal.

	Age	Freq	Threshold	Electrode	Impedance	Threshold		Dynamic Range		eCAP slope	
	(months)	(kHz)	dB SPL	E0 = apicale	(Ohm)	(nCoulomb)		(nC)		(mV/nC)	
						PA	PD	PA	PD	PA	PD
A1	22	2	70	E0	2002	12	18	19.5	13.5	48	39.7
		16	15								
		32	50								
A2	19	2	60	E0	2071	10.5	10.5	21	16.5	61.9	39.9
		16	20								
		32	45								
A3	8	2	40	E0	2412	19.5	15	12	10	78.4	22.9
		16	0								
		32	30								
A4	18	2	60	E0	2503	13.5	16.5	18	7.5	24.4	28.5
		16	-5								
		32	40								
A5	20	2	50	E0	2640	13.5	13.5	18	9	56	70.7
		16	-5								
		32	30								
A6	17	2	55	E0	2776	13.5	18	12	12	39.8	95.5
		16	5								
		32	40								
A7	17	2	65	E0	1809	16.5	12	15	19.5	135.6	186.1
		16	20								
		32	50								
A8	11	2	65	E0	3004	16.5	13.5	15	10.5	70.3	116.1
		16	30								
		32	65								
A9	8	2	55	E0	1570	15	10.5	16.5	21	50.6	38.5
		16	-5								
		32	30								
A10	15	2	50	E0	2587	13.5	13.5	16.5	18	52	53.6
		16	5								
		32	35								

Each column shows from left to right, the animal’s age (months at the time of implantation), the threshold of ABR (dB SPL) for the different frequencies tested (kHz), the period of study (weeks), the location of the stimulating electrode, the impedance measured for the stimulating electrode (Ohm), and for each strategy, the charges at threshold (nC), the size of the dynamic range (nC), and the slope of the eCAP growth function (mV/nC) after linear fitting and selection of the fit with the least residual. For acute animals, impedance measurements given in the table were measured at the beginning of the recording session. The charges at threshold, the dynamic range and the eCAP slope are based on the growth function presented in Fig 5.

In conclusion, individual growth functions differ considerably between animals. This suggests that drawing conclusions based on eCAP group data might not be appropriate for defining the most efficient strategy in terms of threshold and dynamic range in a particular subject.

#### Impact on cortical responses

We decided to take advantage of our acute animals to investigate whether or not central physiological responses are impacted by the difference observed between the 2 strategies on the eCAP growth functions.

**Fig 6:**
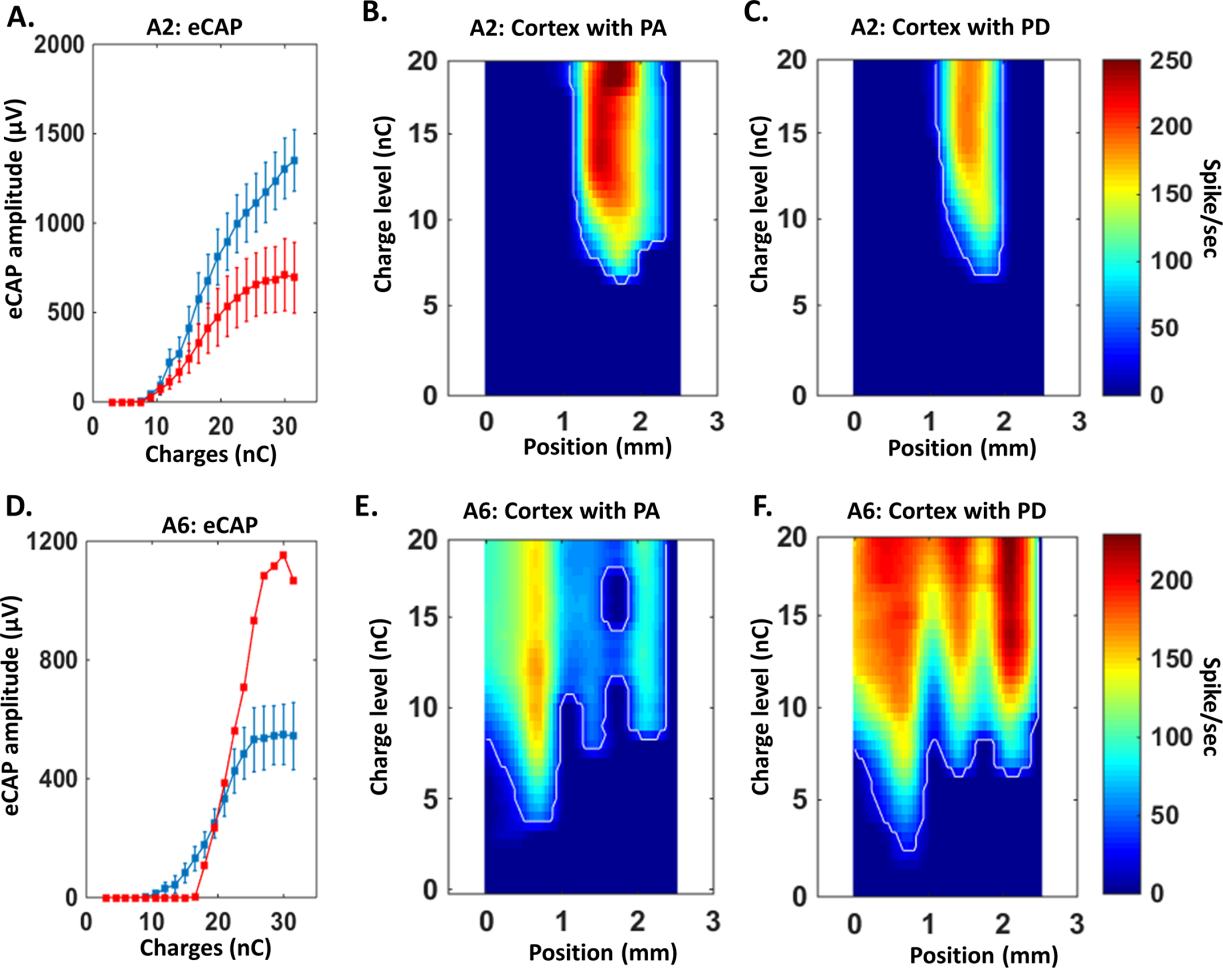
Relationship between eCAP growth function and strength of activation in A1. A. Mean (+SEM) eCAP growth functions in animal A2 for both strategies of stimulation. As showed in Fig 5, this animal has equivalent thresholds and higher eCAP amplitude with the pulse amplitude strategy (PA) than the pulse duration strategy (PD) leading to a broader dynamic range (see Table 1). B-C. Representation of the spatial activation in the auditory cortex for animal A2 when the charge is increased in the pulse amplitude strategy (B) or the pulse duration strategy (C). The zero position corresponds to the first electrode (most caudal) of the cortical array (inter electrode distance = 350μm). The color code represents the firing rate evolution (in spikes/second, blue = lowest; red = highest) as charge increases (defined by 20 levels of charges, see Methods). Consistent with the effect observed on eCAP growth function (A.), the pulse amplitude strategy elicits a stronger response in the auditory cortex than the pulse duration strategy. D. Mean (+SEM) eCAP growth functions of animal A6 for both strategies of stimulation. As showed in Fig 5, this animal has a higher eCAP amplitude with the pulse duration strategy than the pulse amplitude strategy. E-F. Representation of the spatial activation in the auditory cortex for animal A6 when the charge is increased with the pulse amplitude strategy (E) or the pulse duration strategy (F). Consistent with the effect observed on the eCAP growth function (D.), the pulse duration strategy elicits a stronger response in the auditory cortex than the amplitude duration strategy.

In acute animals, neuronal evoked discharges were recorded in the primary auditory cortex. We quantified the strength of the cortical evoked responses and the spatial spread of activation induced by the two strategies in the primary auditory cortex. Fig 6 shows two examples of such quantifications. On the top row, we display the eCAP growth functions for the animal A2 (Fig 6A), the spread of cortical activation triggered by the PA strategy (Fig 6B) and the PD strategy (Fig 6C). Based on eCAP growth functions, the PA strategy triggered larger eCAP amplitudes. Comparisons between Fig 6B and 6C show that the response strengths and the spatial cortical activation were larger with the PA strategy. On the bottom row, we show the eCAP growth functions for animal A6 (Fig 6D), the cortical activation triggered by the PA strategy (Fig 6E) and the PD strategy (Fig 6F). Based on the eCAP growth functions, the PD strategy triggered larger eCAP amplitudes. The comparison between Fig 6E and 6F shows that response strengths were stronger with the PD strategy than with the PA strategy. For this animal, the effect on the spatial activation was modest, probably because in that particular case, both strategies already activated a large fraction of the primary auditory cortex at 15nC.

**Fig 7:**
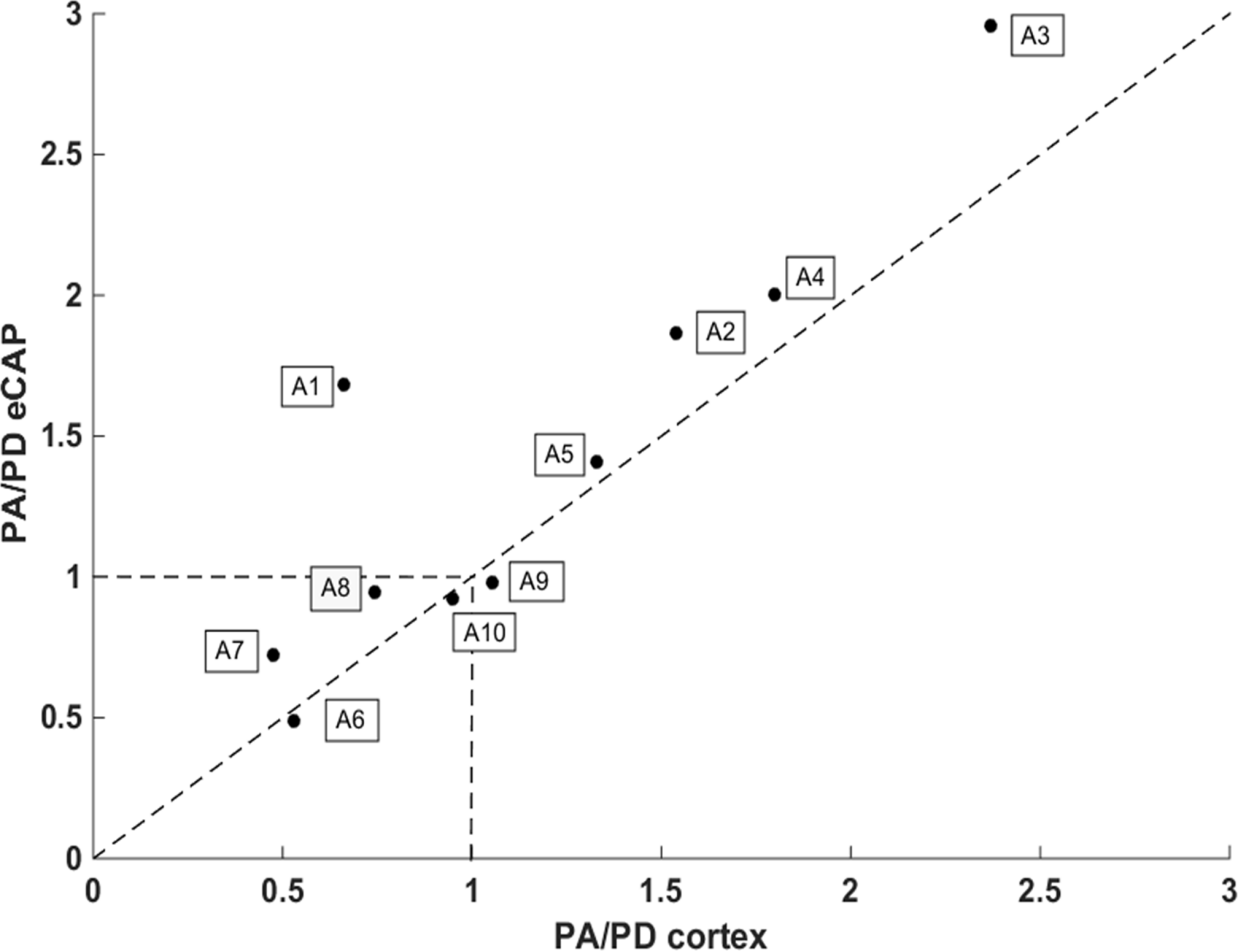
Relationship the PA/PD ratio at the eCAP and cortical level. For each acute animal, the ratio between the eCAP maximum amplitude triggered by the PA and PD strategy was plotted against the ratio between the strength of all cortical responses obtained with the PA and the PD strategy. At the level of the eCAP, the last 3 points of the growth function shown in Fig 5 were considered; at the cortical level, the responses of all the electrodes responding at the 3 last charge levels were pooled together. There was a significant correlation between the values of the PA/PD eCAP and the values of the PA/PD cortex (r = 0.895; p<0.001) indicating that the strategy producing the largest eCAP amplitude, also produced the largest cortical responses.

To quantify these effects, we computed the ratio between the maximum eCAP amplitude triggered by the PA and the PD strategy (PA/PD eCAP) and the ratio between the cortical firing rate triggered by the PA and PD strategy averaged over the responsive electrodes (PA/PD cortex). Fig 7 shows for each acute animal, the ratio PA/PD cortex (abscise) as a function of the ratio PA/PD eCAP (ordinate). For both axes, dots above 1 indicate that the PA strategy triggered larger responses either at the eCAP or cortical level; dots below 1 indicate that the PD strategy triggered larger responses. As shown in Fig 7, for all the animals (except A1) where PA/PD eCAP was above 1, the value of PA/PD cortex was also above 1. For the 2 animals for which the PA/PD eCAP was clearly below 1 (A6, A7), the value of PA/PD cortex was also below 1. Last, in the case of the 3 animals (A8, A9, A10) for which the 2 strategies were equivalent for the final eCAP amplitude, the cortical responses were also equivalent with the two strategies and the dots are around 1 for both eCAP and cortex. As illustrated on Fig 7, many dots are around the diagonal line and there was a significant correlation between the amplitude ratio PA/PD at the level of the eCAP and the amplitude ratio PA/PD in terms of cortical firing rate (r = 0.895; p<0.001). In contrast, there was no correlation between the maximal eCAP amplitude and the spatial extent of cortical activation both with the PA and the PD strategy (R = 0.40 and R = 0.33 respectively).

Thus, for one animal to another, either the PA or the PD strategy was the most efficient in producing larger eCAP amplitudes, and this difference clearly impacted the strength of neuronal responses at the cortical level.

## Discussion

In the present study, eCAPs recorded from either chronically or acutely implanted guinea pigs were compared using two strategies to increase the injected charges (and potentially to increase the sound intensity). Analyzing the eCAP amplitude as a function of the injected charges revealed that the growth functions differed from one animal to another. The differences observed with the two strategies on the eCAP amplitude were also detected in the firing rate of cortical neurons.

### Methodological issues

According to the Ethical 3R rule in animal experimentation, we tested a small number of animals during multiple eCAP recording sessions (see Table 1). In experiment 2, eCAPs were recorded in ten acute animals have been tested 4–8 times during the 3h post hours implantation.

One limitation in the present study comes from the use of monopolar stimulation that is known to produce larger patterns of excitation compared to multipolar stimulation ([2], [3], [4]). Monopolar stimulation is widely used in humans so our choice allows for comparison with clinical research. This mode of stimulation was also used here because it requires lower charges to reach physiological threshold compared to bipolar or tripolar stimulation ([39], [40]). Lower charge levels are advantageous to avoid facial twitching (which can mask electrophysiological responses) and to measure clear responses without generating large current artifacts. In the future, comparing the present results with those obtained with bipolar or tripolar stimulations should be informative especially if a larger number of electrodes can be inserted in the cochlea (by decreasing the diameter of the electrode-array). Here, the current spread associated with monopolar stimulation was not assessed with techniques such as the spread of excitation ([3], [41], [42], [43]) because such techniques cannot be used when a small number of electrodes are inserted in the scala tympani. Lastly, “anodic first” stimulations were used to match human studies, which show that humans are more sensitive to the anodic phase of the pulse ([44], [45], [46], [47], [48]), even if it has been reported in animals, that responses are more sensitive to the cathodic phase of the stimulation [49]. We considered that this should not have a major impact on the comparison between pulse amplitude and pulse duration strategies, and this choice should allow us to extrapolate our results to human studies.

Another issue is that, as neither computed tomography scans nor histology of the cochlea were conducted in our animals, we cannot assess how homogeneous the electrode positions were relative to the modiolus. This might be important because, in theory, the position of the electrode array in the scala tympani has an impact on the threshold values: the closer from the modulus, the lower the threshold. Note that this threshold difference between close and distant electrodes is larger in case of degenerated nerve [50].

### Origins of inter-animal variability

#### In chronic animals

As it is the case with any chronic implant inserted in biological tissue (e.g. see [51], [52]), many factors can explain the differences observed between subjects when testing the eCAP in chronically implanted animals. The first factor is an inflammatory reaction and the potential fibrosis that can considerably differ between subjects. Studies have shown that cochleostomia is the technique that induces the strongest fibrosis response [53] compared with insertion via the round window. Fibrosis goes primarily from the cochleostomy to the tip of the implant ([54], [55]). Recently, Wilk et al. [56] found a correlation between the increase in electrode impedance and the percentage of growth tissue around it, but the impedance in that paper was twice higher than in our case (5 kOhm vs 2–3 kOhm) so this finding might not apply to our data. They also showed that fibrosis was more prominent in the basal turn of the cochlea and can be reduced by coating the implant with dexamethasone making the link with the inflammatory reactions (see also [57]).

As previously explained, eCAP amplitudes are unreliable during the first week after implantation [37]. It is generally assumed that the reduction in eCAP amplitudes (until they stabilize during the second week post-surgery) is the result of tissue growth around the electrodes which promotes the increase in electrode impedance (see for example [58] for discussion about impedance changes taking place after implantation). Several factors might explain the unstability of the eCAP during the first week post-implantation. First, cochleostomy always induces a substantial loss of perilymphatic liquid, which can reduce the efficiency of electrical stimulations, leading to smaller eCAP responses. Then, the development of fibrosis can prevent leakage of the perilymphatic liquid, allowing normal volumes of perilymph and, as a consequence bring the efficiency of stimulations back to normal. This means that over time, changes of fluid volumes and their compositions can occur and can differ between subjects depending on the size to the cochleostomy and the growth of fibrosis. The extent to which these factors explain inter-subject variability remains unknown. However, it would be of interest to evaluate whether the use of dexamethasone (or other anti-inflammatory treatment) could reduce the shift of eCAP amplitudes over time and/or inter-subject variability [56, 57].

We then decided to perform experiments in acute animals in conditions where the inflammatory reactions and the fibrosis are not yet present.

#### In acute animals

Several events might be involved in inter-subject variability in acute animals. Previous studies envisioned a relationship between the strength of electrophysiological responses when changing stimulation parameters and the level of degeneration of nerve fibers [23]. More precisely, it seems that the responses to the change in pulse duration are more correlated with nerve degeneration than responses to change in pulse amplitude [59]. One can propose that the differences between animals, i.e. the efficiency of a given strategy over another could be linked with the status of the nerve fibers at the time of implantation. Without histological results it is not possible to evaluate in these experiments whether the eCAP growth functions induced by the two strategies are related to the health of the nerve. However, as stated in the results, the lowest electrical thresholds and the largest dynamic ranges were not associated with the lowest acoustic threshold before implantation.

Our eCAP results question the hypothesis of the “equal charge, equal loudness” theory that was already put into doubt by Zeng et al. [16]. In this study, an increment in stimulus amplitude produced a significantly louder sensation than the same change associated with stimulus duration, suggesting a potential difference in the way pulse duration and pulse amplitude are integrated in the auditory system. Other factors could also be involved in the inter-subject variability. The potential loss of perilymphatic fluid could have much more influence on eCAP responses in acute preparations compared with chronic implants, as well as the position of the electrode array in the scala tympani. However, it is not clear why this factor would affect more the amplitude of eCAP responses induced by the pulse amplitude rather than induced by the pulse duration strategy.

## Conclusions and clinical implications

Some human studies have suggested that proposing a generic solution for implant coding might not be realistic. For example, Chua et al. [60] tried four different strategies for coding sound loudness and measured the number of discriminable loudness steps in individual patients. For each subject, it was possible to find a particular strategy that was better than others because it allowed a larger “number of loudness steps”, but there was no group tendency, i.e., none of the strategies was able to promote a larger number of loudness steps in every subject. The physiological basis for the perceptual differences induced by different strategies of stimulation remains unknown. Potentially, eCAP measures could be a way to link perceptual performance and physiological effects of the stimulation. However, human studies have sometimes questioned whether the use of eCAP provides sufficiently objective measures for programming cochlear implants, mainly because there is a low correlation between eCAP thresholds and the behavioral thresholds when tested at high rates of stimulation ([38], [61]). In fact, when increasing the rate of stimulation, the average changes in behavioral threshold can be predicted when using the average eCAP data, but individual variations in slope of the threshold vs. rate function is not a good predictor of individual variations in eCAP [38].

One limitation of the present study is that has been conducted in animals exhibiting modest hearing loss before implantation. In contrast, in human patient candidate for cochlear implantation, the degree of hearing loss is high and the period of sensory deprivation before implantation can be quite long. Thus, it is not entirely certain that the results obtained here can apply to situations where some auditory nerve fibers are missing. Even if it is quite difficult to envision that the loss of nerve fibers will impact more one strategy than another, animal models of hearing deficits (e.g. see [23], [62], [63]) should be used to confirm our results.

The results derived from the eCAP growth function suggest that between subjects, either the pulse amplitude or the pulse duration was the most efficient strategy to produce the largest eCAP amplitude. It was therefore legitimate to look for central correlates of the difference in eCAP growth functions induced by the two strategies. We find a clear relationship between the effects of the strategy on the eCAP amplitude and the strength of cortical activation. Clearly, stronger cortical activation in the primary auditory cortex is not necessarily a key factor for explaining the perceptive performance of human subjects. Considerable research efforts are still required to understand why, in a given patient some stimulation strategies, or parameters, are more efficient than others at the perceptual level. More generally, rather than adjusting a fixed pre-existing strategy used by a given manufacturer, it might be beneficial in human subjects to have access to all potential parameters involved in coding a specific sound feature (such as the sound intensity) and to select those that are more efficient on an individual basis.

## Supporting information

S1 Fig Examples of eCAP acquisition and quantification. A. Raw traces of eCAP recorded from animal A1 by increasing the pulse amplitude from blue (lowest value) to red (highest value). Each curve corresponds to the mean response averaged over 128 stimulations after removing the stimulation artifact (see Methods). The circles indicate the minimal (green) and maximal (red) values detected on each curve by the peak-tracking algorithm (see Methods).
B-C. Amplitudes of the N1-P2 (B) and P2-N2 wave (C) as a function of the stimulation intensity (pulse amplitude in blue and pulse duration in red). Note that the growth functions are similar for the N1-P2 and for the P2-N2 waves.
D-E. Latency of the N1 trough (D) and of the P2 peak (E) as a function of the stimulation intensity (pulse amplitude in blue and pulse duration in red).
(TIF)
