# Supplementary figures and images for: ECAP growth function to increasing pulse amplitude or pulse duration demonstrates large inter-animal variability that is reflected in auditory cortex of the guinea pig
Source: PLoS One. 2018 Aug 2;13(8):e0201771. doi: 10.1371/journal.pone.0201771 (PMC6072127; doi:10.1371/journal.pone.0201771)

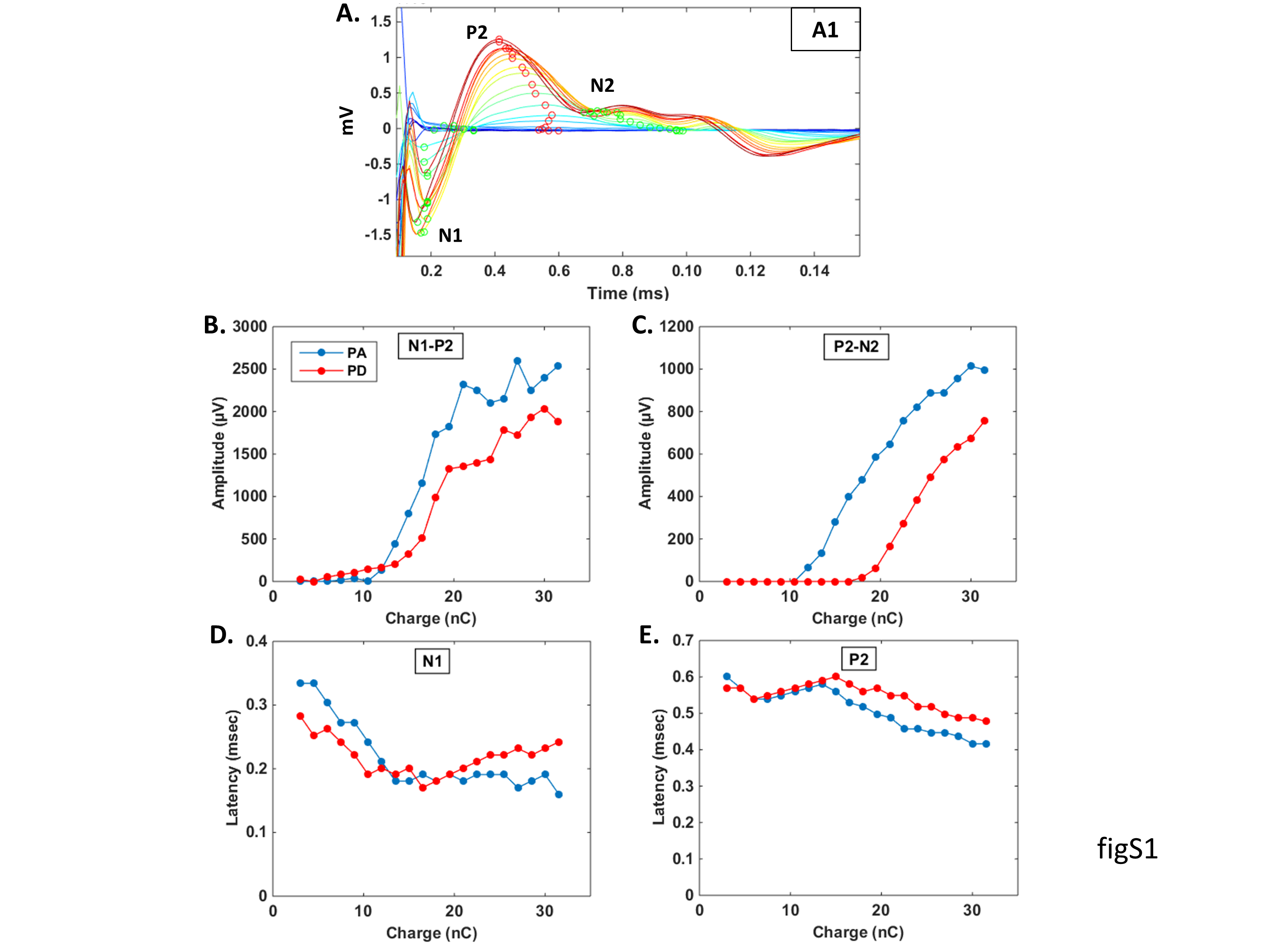

Supplement: S1 Fig — A. Raw traces of eCAP recorded from animal A1 by increasing the pulse amplitude from blue (lowest value) to red (highest value). Each curve corresponds to the mean response averaged over 128 stimulations after removing the stimulation artifact (see Methods). The circles indicate the minimal (green) and maximal (red) values detected on each curve by the peak-tracking algorithm (see Methods). B-C. Amplitudes of the N1-P2 (B) and P2-N2 wave (C) as a function of the stimulation intensity (pulse amplitude in blue and pulse duration in red). Note that the growth functions are similar for the N1-P2 and for the P2-N2 waves. D-E. Latency of the N1 trough (D) and of the P2 peak (E) as a function of the stimulation intensity (pulse amplitude in blue and pulse duration in red). (TIF) [file pone.0201771.s001.TIF]
